# Sex difference in the association of metabolic syndrome with high sensitivity C-reactive protein in a Taiwanese population

**DOI:** 10.1186/1471-2458-10-429

**Published:** 2010-07-21

**Authors:** Ming-May Lai, Chia-Ing Li, Sharon LR Kardia, Chiu-Shong Liu, Wen-Yuan Lin, Yih-Dar Lee, Pei-Chia Chang, Cheng-Chieh Lin, Tsai-Chung Li

**Affiliations:** 1 Department of Family Medicine, China Medical University & Hospital, Taichung, Taiwan; 2 Department of Family Medicine, College of Medicine, China Medical University & Hospital, Taichung, Taiwan; 3 Medical Research, China Medical University & Hospital, Taichung, Taiwan; 4 Department of Epidemiology, University of Michigan, Ann Arbor, Michigan; 5 Department of Psychiatric, Medical College, National Cheng-Kung University, Tainan, Taiwan; 6 Bristol-Myers Squibb (Taiwan) Ltd, Global Development & Medical Affair, Tainan, Taiwan; 7 Administration Center, China Medical University & Hospital, Taichung, Taiwan; 8 Institute of Health Care Administration, College of Health Science, Asia University, Taichung, Taiwan; 9 School and Graduate Institute of Health Care Administration, College of Public Health, China Medical University & Hospital, Taichung, Taiwan; 10 Graduate Institute of Biostatistics & Chinese Medicine Science, China Medical University & Hospital, Taichung, Taiwan; 11 Biostatistics Center, China Medical University & Hospital, Taichung, Taiwan

## Abstract

**Background:**

Although sex differences have been reported for associations between components of metabolic syndrome and inflammation, the question of whether there is an effect modification by sex in the association between inflammation and metabolic syndrome has not been investigated in detail. Therefore, the aim of this study was to compare associations of high sensitivity C-creative protein (hs-CRP) with metabolic syndrome and its components between men and women.

**Methods:**

A total of 1,305 subjects aged 40 years and over were recruited in 2004 in a metropolitan city in Taiwan. The biochemical indices, such as hs-CRP, fasting glucose levels, lipid profiles, urinary albumin, urinary creatinine and anthropometric indices, were measured. Metabolic syndrome was defined using the American Heart Association and the National Heart, lung and Blood Institute (AHA/NHLBI) definition. The relationship between metabolic syndrome and hs-CRP was examined using multivariate logistic regression analysis.

**Results:**

After adjustment for age and lifestyle factors including smoking, and alcohol intake, elevated concentrations of hs-CRP showed a stronger association with metabolic syndrome in women (odds ratio comparing tertile extremes 4.80 [95% CI: 3.31-6.97]) than in men (2.30 [1.65-3.21]). The p value for the sex interaction was 0.002. All components were more strongly associated with metabolic syndrome in women than in men, and all sex interactions were significant except for hypertension.

**Conclusions:**

Our data suggest that inflammatory processes may be of particular importance in the pathogenesis of metabolic syndrome in women.

## Background

The metabolic syndrome is a constellation of interrelated factors of metabolic origin including abdominal obesity, high blood pressure, a low level of high-density lipoprotein (HDL) cholesterol, a high triglyceride level, and a high plasma glucose concentration [1-3]. The etiology of this syndrome is largely unknown but presumably represents a complex interaction between genetic, metabolic, and environmental factors [4,5]. Individuals with these characteristics commonly manifest a prothrombotic and proinflammatory state, which is associated with an increased risk for myocardial infarction, stroke and incident type 2 diabetes [6-18]. Although previous cross-sectional studies show that elevated high sensitivity C-reactive protein (hs-CRP) levels correlate significantly with features of the metabolic syndrome, including adiposity, hyperinsulinemia, insulin resistance, hypertriglyceridemia, and low HDL cholesterol [19,20], most of them examined the relationship between hs-CRP and individual components of metabolic syndrome. The previous study indicated that hs-CRP levels were higher in women compared with men and this gender difference was maintained across all ethnic subgroups [21]. In addition, sex differences have been reported for associations between components of metabolic syndrome and inflammation, the question of whether there is an effect modification by sex in the association between inflammation and metabolic syndrome has not been investigated in detail. Therefore, in this community-based cross-sectional study, we examined whether the relationship between hs-CRP and the metabolic syndrome, defined by the American Heart Association and the National Heart, Lung, and Blood Institute (AHA/NHLBI, 2005) statement [22], is modified by gender, obesity, microalbuminuria, insulin resistance, cardiovascular risk, and peripheral vascular disease (PVD) in a Taiwanese population aged forty years and over.

## Methods

### Participants

This was a community-based cross-sectional study based on data from Taichung Community Health Study (TCHS). A total of 2,359 residents aged 40 and over in Taichung City, Taiwan, participated in October 2004. A two-stage sampling design was used, with a sampling rate proportional to size within each stage. At each stage, simple random sampling was used. In the first stage of sampling, the sampling unit was Li (administrative units) and the selection probability for Li was set at 0.125. A total of 39 Lis were randomly selected from 8 city districts. In the second stage, 110 individuals were randomly selected from each sample Li. A total of 4280 individuals were selected and 750 individuals who were not eligible were excluded. A total of 3,530 subjects were eligible, and 2,359 agreed to participate with an overall response rate of 66.83%. The detailed methodology has been described elsewhere [23-27]. Levels of hs-CRP only were measured in the first 1305 subjects. This study was approved by the Human Research Committee of China Medical University Hospital. Written informed consent was obtained from each participant.

### Anthropometric measurement and laboratory examination

Anthropometric measurements and blood samples were obtained from the complete physical examination. Weight and height were measured on an autoanthropometer (super-view, HW-666), with the subjects shoeless and wearing light clothing. Body mass index (BMI) was derived from the formula of weight (kg) ÷ (height)^2 ^(m^2^). With the participant standing, waist circumference was measured midway between the superior iliac crest and the costal margin, and hip circumference at its maximum protrusion point of the buttocks around the pelvis, and then the waist-to-hip ratio (WHR) was calculated as a measure of regional fat distribution. Percent body fat mass (%FM) was assessed by conventional tetrapolar bioelectrical impedance analysis using the Tanita BC-418 MA Impedanciometer (Tanita Corp., Tokyo, Japan) [28]. Blood pressure was measured by an electronic device (COLIN, VP-1000, Japan) three times after the subjects were at rest for 20 minutes. The lowest systolic and diastolic blood pressure was recorded.

Blood was drawn from an antecubital vein in the morning after a 12-hour overnight fasting and was sent for analysis within four hours of blood collection. Biochemical markers such as fasting plasma glucose, high-density lipoprotein cholesterol (HDL-C), and triglyceride were analyzed by a biochemical autoanalyzer (Beckman Coluter Synchron system, Lx-20, Fullerton, CA, USA) at the Clinical Laboratory Department of China Medical University Hospital. Plasma cholesterol and triglyceride levels were determined by an enzymatic colorimetric method. The HDL-C level was measured by a direct HDL-C method; HDL lipoprotein particles were solubilized by a detergent to release HDL-C that reacted with cholesterol esterase and cholesterol oxidase in the presence of chromogens to produce a colored product. The low-density lipoprotein cholesterol (LDL-C) level was measured by a direct LDL-C method, which used 2 kinds of detergents to solubilize the LDL particles. The serum insulin level was measured by a commercial enzyme-linked immunosorbent assay kit (Diagnostic Products, Los Angeles, CA). The interassay coefficient of variation (CV) for insulin was 8.7% and the intra-assay CV was 3.4%. Insulin sensitivity was estimated with a Homeostasis Model Assessment (HOMA-IR) equation. The HOMA-IR equals fasting serum insulin (μU/ml) times fasting plasma glucose (mmol/l) divided by 22.5 [29]. Hs-CRP levels were measured by nephelometry, a latex particle-enhanced immunoassay (TBA-200FR, Tokyo, Japan). The interassay and intraassay CVs were < 2.0% and < 1.9%, respectively. The lower detection limit of the assay was 0.1 mg/l. Urinary creatinine (Jaffe's kinetic method) and albumin (colorimetyl bromcresol purple) were measured by an autoanalyzer (Beckman Coluter Synchron system, Lx-20, Fullerton, CA, USA). The interassay precision coefficient of variation was < 3.0% for both creatinine and albumin concentrations. The urinary albumin-to-creatinine ratio (ACR) in the morning urine sample was used as a marker of the albumin excretion rate. Urinary ACR ranging from 30 mg g-1 creatinine to 300 mg g-1 creatinine was defined as microalbuminuria [30].

The measurement of brachial-ankle pulse wave velocity (baPWV) and the ankle-brachial index (ABI) were determined using an automatic waveform analyzer (VP-1000; Colin Co., Komaki, Japan) with well-documented validity and reproducibility (coefficient of variation [CV] = 3.31% and reproducibility coefficient = 0.947) [31]. Higher baPWV values indicated more severe arterial stiffness. Lower ABI values indicated more severe PVD. High baPWV was defined as a value higher than 1,400 cm/s, whereas an ABI index < 0.9 was considered the presence of PVD [32].

Using the Framingham risk score based on the LDL-C level [33], the estimated total coronary heart disease risk over a 10-year period for every individual was calculated. Data on sociodemographic characteristics, including gender, age, smoking, drinking, betel nut chewing (the fourth most widely used addictive substance in the world that were associated with metabolic syndrome, chronic kidney disease, and heart disease [34-37]), family history of cardiovascular-related diseases, physician-diagnosed diseases, and medication history were collected when the participants underwent a complete physical examination.

### Definition of metabolic syndrome

The AHA/NHLBI statement was used to define the metabolic syndrome, which had three or more of the following abnormality: elevated waist circumference, elevated triglycerides (≧150 mg/dL), reduced HDL-C (< 40 mg/dL for men, < 50 mg/dL for women), elevated blood pressure (BP≧130/≧85 mmHg), and elevated fasting glucose (≧100 mg/dL). Elevated waist circumference was defined by Asia-Pacific cutoff limits: waist circumference of 90 cm or more for men and 80 cm or more for women because the importance of ethnic-specific cutoff points for waist circumference [38]. As stipulated by the definition we included all individuals receiving pharmacologic treatment for hypertension as having elevated blood pressure, all subjects receiving fibrates as possessing both elevated triglycerides and reduced HDL-C, and all subjects previously diagnosed with type 2 diabetes as having elevated fasting glucose.

### Statistical analysis

Continuous variables were reported as mean, whereas categorical variables were reported as number (percentage). Chi-square test was used to analyze the statistical differences in cardiovascular risk factors between participants according to hs-CRP tertiles of gender. Since the distribution of hs-CRP levels was skewed to the right, natural log-transformation for CRP (ln hs-CRP) was used to normalize the data. The comparisons of mean values of ln hs-CRP (geometric means of hs-CRP) according to the number of metabolic disorders were performed by analysis of variance. Using a general linear model, we tested the linear trends for increasing geometric means of hs-CRP levels across the number of MetS's components. For ease of interpretation, geometric means of un-transformed hs-CRP values and their standard deviations were reported. Multiple logistic regression analysis was used to investigate the effect of hs-CRP as sex-specific tertiles on metabolic syndrome, after adjusting for age, smoking, alcohol drinking, and betel nut chewing status, an interaction of hs-CRP and sex was further examined by adding the interaction term into the full model. The cutoff points of hs-CRP tertiles for males were 0.100 and 0.157, and for females were 0.1 and 0.163, respectively. The high risk groups for %FM and Framingham risk scores were determined by the upper quartile of their distributions. All analysis was conducted using SAS version 9.1 (SAS Institute Inc, Cary, NC). Odds ratios and 95% confidence intervals were calculated. A significant level of p < 0.05 was reported.

## Results

A total of 1291 subjects (625 men and 666 women) were analyzed in the final model. The distributions of cardiovascular risk factors according to groups of the tertile for hs-CRP, stratified by gender, are shown in Table 1. The mean values of BMI, waist circumference, waist-to-hip ratio, fasting blood glucose, fasting insulin, HOMA-IR, diastolic and systolic blood pressure, and %FM significantly increased from the lowest tertile to the highest tertile of hs-CRP in both sexes, but the mean values of HDL-C decreased. The prevalence of arterial stiffness, microalbuminuria, and high Framingham risk score also significantly increased from the lowest tertile to the highest tertile of hs-CRP in both sexes.

**Table 1:** The distributions of cardiovascular risk factors according to groups of the tertile for hs-CRP, stratified by gender

Variables	Mean (SD)							
	
	Men (N = 625)				Women (N = 666)			
	Lower tertile ≦0.1 mg/l (N = 299)	Middle tertile 0.1-0.157 mg/l (N = 123)	Upper tertile > 0.157 mg/l (N = 203)	P value	Lower tertile ≦0.1 mg/l (N = 330)	Middle tertile 0.1-0.163 mg/l (N = 115)	Upper tertile > 0.163 mg/l (N = 221)	P value
Age (years)	56.8 (11.6)	57.5 (12.4)	59.0 (13.0)	0.15	52.2 (9.4)	55.8 (10.8)	56.7 (9.7)	< .001
Body mass index (kg/m^2^)	24.4 (2.9)	25.4 (3.3)	25.3 (3.2)	< .001	22.6 (2.5)	24.4 (3.2)	25.3 (3.7)	< .001
Smoking (%)^†^	74 (24.8)	31 (25.2)	70 (34.5)	0.04	12 (3.7)	1 (0.9)	10 (4.5)	0.21
Drinking (%)^†^	120 (40.1)	55 (44.7)	66 (32.5)	0.07	46 (14.0)	7 (6.1)	19 (8.6)	0.03
Betel nut chewing (%)^†^	14 (4.7)	12 (9.8)	13 (6.5)	0.15	1 (0.3)	0 (0.0)	0 (0.0)	0.60
Exercise (%)^†^	201 (67.5)	85 (69.1)	131 (64.5)	0.66	224 (67.9)	77 (67.0)	136 (61.5)	0.29
Waist circumference (cm)	85.1 (8.2)	88.2 (7.8)	88.3 (9.1)	< .001	73.6 (6.5)	78.3 (8.1)	81.3 (9.6)	< .001
Waist-to-hip ratio	0.9 (0.1)	0.90 (0.1)	0.90 (0.1)	0.004	0.79 (0.1)	0.82 (0.1)	0.83 (0.1)	< .001
Fasting blood glucose (mg/dl)	102.8 (24.2)	103.5 (22.4)	111.3 (33.5)	0.002	96.52 (21.3)	99.2 (18.1)	109.1 (37.1)	< .001
Fasting insulin (uU/ml)	8.1 (6.1)	9.5 (7.0)	10.0 (7.4)	0.005	6.5 (5.7)	8.1 (7.3)	10.1 (7.4)	< .001
HOMA-IR	2.1 (1.9)	2.5 (2.2)	2.8 (2.3)	0.001	1.6 (1.8)	2.1 (2.3)	2.9 (2.8)	< .001
Total cholesterol (mg/dl)	202.6 (35.3)	201.2 (37.7)	204.5 (39.7)	0.73	205.0 (40.0)	214.0 (33.5)	207.4 (37.5)	0.10
Triglyceride (mg/dl)	121.2 (105.1)	143.9 (41.2)	139.1 (74.2)	0.05	84.3 (45.3)	107.7 (53.4)	127.2 (88.7)	< .001
HDL-cholesterol (mg/dl)	42.7 (11.7)	41.2 (11.1)	39.9 (9.6)	0.02	53.7 (14.0)	50.4 (12.9)	46.6 (10.8)	< .001
LDL-cholesterol (mg/dl)	129.4 (31.7)	127.0 (33.9)	133.5 (36.3)	0.20	125.3 (34.8)	134.6 (27.8)	129.9 (36.2)	0.03
Diastolic blood pressure (mmHg)	80.2 (10.4)	83.0 (10.4)	83.4 (11.3)	0.002	72.7 (11.2)	76.3 (13.6)	77.8 (11.6)	< .001
Systolic blood pressure (mmHg)	133.2 (19.1)	138.1 (19.1)	140.6 (19.9)	< .001	125.6 (18.6)	134.1 (22.7)	136.8 (22.2)	< .001
Family history of diabetes (%)^†^	80 (26.8)	29 (23.6)	47 (23.3)	0.62	93 (28.2)	19 (16.5)	59 (26.7)	0.04
Diabetes (%)^†^	35 (11.7)	12 (10.2)	43 (20.7)	0.006	17 (5.2)	14 (11.6)	41 (19.1)	< 0.001
Antidiabetes medication (%)^†^	26 (8.7)	5 (4.2)	30 (14.4)	0.031	12 (3.6)	5 (4.1)	26 (12.1)	< 0.001
Antihypertension medication (%)^†^	47 (16.0)	31 (27.0)	61 (29.6)	< 0.001	39 (12.0)	26 (21.9)	58 (27.2)	< 0.001
Antihyperlipid medication (%)^†^	17 (5.9)	8 (7.0)	22 (10.8)	0.126	11 (3.4)	6 (5.1)	13 (6.2)	0.296
Metabolic syndrome (%)^†^	106 (35.5)	51 (43.2)	116 (55.8)	< 0.001	56 (17.0)	55 (45.5)	112 (52.1)	< 0.001
Arterial stiffness ^†^	16 (5.4)	6 (4.9)	15 (7.4)	0.55	24 (7.3)	7 (6.1)	18 (8.1)	0.79
PVD ^†^	198 (67.1)	85 (65.1)	165 (86.8)	0.001	155 (47.4)	68 (59.1)	155 (71.1)	< .001
Microalbuminuria (ACR≧30 *μ*g/min)^†^	45 (15.1)	21 (17.1)	52 (25.6)	0.01	52 (15.8)	36 (31.3)	68 (31.2)	< .001
High Framingham risk factors (%)^†^	76 (25.4)	37 (30.1)	79 (43.8)	< .001	79 (23.9)	45 (39.1)	117 (52.9)	< .001
%body fat mass	25.1 (5.0)	27.1 (5.8)	28.1 (5.5)	< .001	34.2 (5.2)	37.8 (4.9)	39.1 (5.9)	< .001

Homeostasis model assessment of insulin resistance (HOMA-IR), high-density lipoprotein (HDL), low-density lipoprotein (LDL), albumin-creatinine ratio (ACR), ankle-brachial index (ABI), brachial-ankle pulse wave velocity (baPWV), highly sensitive C-reactive protein (hs-CRP), peripheral vascular disease (PVD): ankle-brachial index (ABI) < 0.9; arterial stiffness: brachial-ankle pulse wave velocity (baPWV) > 1400 cm/s; Framingham risk factors^33^, ^†^: n (%)

**Figure 1:**
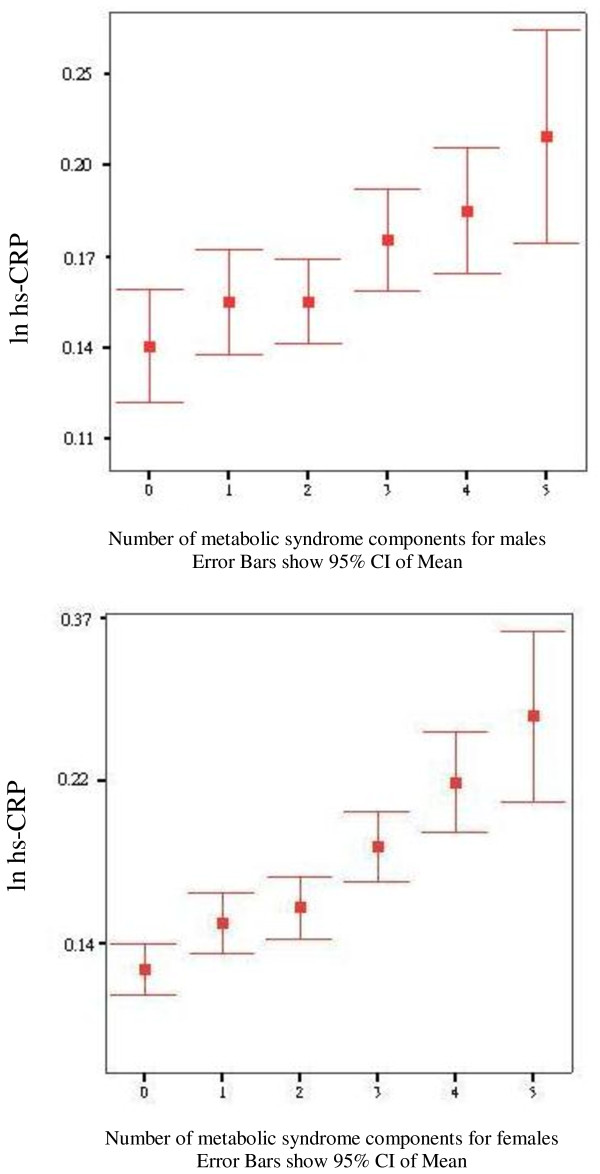
Figure 1 Geometric means of Hs-CRP and 95% CI according to the number of abnormal components of the metabolic syndrome, defined by criteria of the American Heart Association/National Heart, Lung, and Blood Institute (AHA/NHLBI) according to gender.

Fig 1 shows the geometric means of hs-CRP by the number of components of the metabolic syndrome. There was a linear increase in geometric means of hs-CRP with the increasing number of components of the metabolic syndrome in both sexes (P for trend < 0.001 for both men and women). CRP levels with 0-3 components of MS are similar in men and women but are raised more with each additional MS component in women compared to men (p = 0.018 for sex interaction).

After adjusting for age, smoking status, alcohol drinking, and betel nut chewing, hs-CRP was associated with the metabolic syndrome defined by AHA/NHLBI and all its components' abnormalities (Table 2). The ORs for women were greater than those for men. In men, the OR of the middle tertile compared to the lowest tertile was significant only for abdominal obesity. The ORs of upper tertile were significant for all components of metabolic syndrome except for low HDL-C. In women, the ORs of middle tertile were significant for hypertriglyceridemia, abdominal obesity, and low HDL-C and those for the upper tertile were significant for all components. In both sexes, the linear trend was statistically significant for metabolic syndrome and all its components except for low HDL-C in men. After either excluding those individuals with diabetes and fibrate users or focusing on nonsmokers, similar findings were found. Sex interactions were significant for metabolic syndrome and all components except for hypertension (p = 0.002 for sex*metabolic syndrome; p = 0.049 for sex*hyperglycemia; p = 0.004 for sex*hypertriglyceridemia; p < 0.001 for sex*abdominal obesity; and p = 0.017 for sex*low HDL-C).

**Table 2:** The adjusted odds ratios (ORs) of having metabolic syndrome and abnormalities of its components for tertiles of hs-CRP

Variables	Adjusted OR (95% CI) ^a^					
	
	Men		P for	Women		P for
	Middle tertile (0.1-0.157 mg/l)	Upper tertile (> 0.157 mg/l)	trend	Middle tertile (0.1-0.163 mg/l)	Upper tertile (> 0.163 mg/l)	trend
Metabolic Syndrome (AHA/NHLBI)	1.60 (1.11-2.31)	2.30 (1.65-3.21)	< .0001	3.52 (2.28-5.42)	4.80 (3.31-6.97)	< .0001
Hyperglycemia	0.97 (0.67-1.41)	1.46 (1.05-2.04)	0.03	1.37 (0.91-2.07)	1.81 (1.28-2.55)	0.0008
Hypertriglyceridemia	1.44 (0.97-2.12)	1.93 (1.36-2.74)	0.0002	2.00 (1.22-3.29)	3.58 (2.38-5.39)	< .0001
Hypertension (%)^†^	1.19 (0.80-1.77)	1.77 (1.22-2.57)	0.003	1.06 (0.70-1.60)	2.00 (1.41-2.83)	0.0001
Abdominal obesity (%)^†^	1.50 (1.00-2.23)	1.89 (1.32-2.69)	0.0004	3.26 (2.10-5.06)	4.99 (3.41-7.29)	< .0001
Low HDL cholesterol	1.38 (0.96-1.99)	1.39 (1.00-1.94)	0.04	1.54 (1.07-2.23)	2.39 (1.73-3.30)	< .0001

^a^Logistic regression adjusting for age, smoking, alcohol drinking and betel nut chewing.

American Heart Association and National Heart Lung and Blood Institute (AHA/NHLBI)

Table 3 shows the adjusted ORs of metabolic syndrome associated with a 1-SD increase in ln hs-CRP stratified by the status of insulin resistance, obesity, microalbuminuria, cardiovascular risk, and arteriosclerosis for men and women in order to examine the gender difference in the association of metabolic syndrome with hs-CRP according to the status of the above cardiovascular factors. The gender difference still remains the same: adjusted ORs slightly larger in females than in males and more ORs for females than for males reached statistical significance. Due to small sample size in some subgroups, some interactions were not statistically significant.

**Table 3:** The ORs of MetS associated with a 1-SD increase in ln hs-CRP stratified by the status of obesity, microalbuminuria, insulin resistance, cardiovascular risk, and arteriosclerosis for men and women

	P for interaction of sex and hs-CRP	Adjusted OR (95% CI) ^a^	
		
Variables		Men	Women
Insulin resistance (HOMA-IR≧2.53)			
No	0.031	1.29 (1.07-1.55)**	1.19 (0.96-1.47)
Yes	0.057	1.26 (0.98-1.65)	1.93 (1.40-2.78)***
Insulin resistance (insulin≧10.40 μU/ml)			
No	0.081	1.26 (1.04-1.52)*	1.40 (1.11-1.76)**
Yes	0.043	1.26 (0.94-1.75)	1.57 (1.09-2.34)*
High % fat mass			
No	0.073	1.26 (1.07-1.49)**	1.35 (1.10-1.65)**
Yes	0.026	1.10 (0.78-1.60)	1.58 (1.15-2.25)**
Microalbuminuria (ACR≧30 *μ*g/min)			
No	0.072	1.28 (1.08-1.53)**	1.48 (1.21-1.81)***
Yes	0.043	1.29 (0.99-1.72)	1.83 (1.35-2.58)***
High Framingham risk factors			
No	0.042	1.24 (1.03-1.50)*	1.39 (1.12-1.73)**
Yes	0.053	1.33 (1.05-1.72)*	1.82 (1.35-2.54)***
Arterial stiffness			
No	0.115	1.21 (0.84-1.71)	1.51 (1.07-2.12)*
Yes	0.034	1.27 (1.09-1.50)**	1.58 (1.29-1.95)***

^a^Logistic regression adjusting for age, smoking, alcohol drinking and betel chewing.

Framingham risk factors^33^

*:p < 0.05; **:p < 0.01; ***:p < 0.001.

## Discussion

The findings in this population-based study showed that Taiwanese individuals with elevated hs-CRP levels, predominantly within the reference range, had a significantly increased likelihood of having the metabolic syndrome in men and women, independent of their age, smoking, alcohol drinking and betel nut chewing (Table 2). However, associations were considerably stronger in women than in men. In addition, the results showed that hs-CRP was a significant risk factor of metabolic syndrome's components with a stronger association in women. We also found significant gender difference in the association of metabolic syndrome with hs-CRP after stratification according to the status of insulin resistance, obesity, microalbuminuria, cardiovascular risk, and arteriosclerosis (Table 3).

Despite evidence indicating that inflammation is related to metabolic syndrome [20,39], data from epidemiologic studies are sparse. Yudkin et al. examined relationships between levels of hs-CRP and components of metabolic syndrome such as obesity, blood pressure, dyslipidemia and insulin resistance in a group of 107 healthy persons [20]. However, their study focused on exploring the mechanisms underlying the relationship of inflammatory process and coronary heart disease and their finding require further testing in both epidemiological and clinical studies with larger sample size. In Laaksonen's study, they examined the association between CRP levels and the development of the metabolic syndrome and diabetes in 680 men with 11 years of follow-up [39]. In Ridker work, although the relationship of hs-CRP with components of the metabolic syndrome was examined in a large-scale population [40], their study subjects restricted to healthy women, who participating in an ongoing trial of aspirin and vitamin E for primary prevention. Therefore both studies cannot examine the interaction of sex and CRP.

There were two studies examining the association between CRP and metabolic syndrome in Chinese [41,42]. Both of these two studies measuring CRP, not hs-CRP, thus they could not detect CRP levels less than 0.25 mg/L. The median value of CRP in our study was more close to that reported by Chien's et al and much lower than that by Ye et al. Chien's study adopted both the updated WHO and NCEP-ATPIII definition of metabolic syndrome for Asian Americans and Ye's study adopted the NCEP-ATPIII definition while we adopted AHA/NHLBI one. Both of them identified significant association between CRP and metabolic syndrome and/or its components, but they did not examine whether there existed gender difference in this association.

There were limited studies examining whether there exists a sex difference in the association between a proinflammatory state and metabolic syndrome [43]. Our observation that hs-CRP is more strongly associated with metabolic syndrome in women than in men is in line with results of Ahonen's study [43], which included subjects with elevated blood pressure. In addition, their results showed there was no gender difference in the level of hs-CRP in individuals without metabolic syndrome, but the level of hs-CRP was significantly lower in men with metabolic syndrome than in women with metabolic syndrome. In a study observing no gender difference in CRP level [42], an association between CRP and metabolic syndrome had been reported as a whole and gender difference in the association between CRP and metabolic syndrome had not been explored.

Hs-CRP was an easily measured inflammatory biomarker and has several direct effects at the level of the vessel wall [44]. It has been shown hs-CRP has associations with endothelial dysfunction and insulin resistance syndrome [20]. Our finding showed that sex difference in the association of metabolic syndrome with hs-CRP might suggest that hormone might play a role on the inflammatory mechanism. Whether sex hormone is responsible for the association of metabolic syndrome with hs-CRP needs to be explored.

Limitations of this study must be considered. The principal limitation relevant to the interpretation of our results is the use of cross-sectional data; thus, causal pathways underlying the observed relationships cannot be inferred. Second, these analyses were restricted to the first 1305 subjects entering the current study, thus potential selection bias might exist. To assess this possibility, we examined the demographic characteristics of the individuals with and without hs-CRP measurement by comparing age, sex, and administrative unit, and similar distributions were found. The non-differential distributions in age, sex, and administrative unit, indicate this kind of selection error might be random, thus, the results could be biased toward the null, a lesser threat to validity.

This finding would have implications for prevention. AHA/NHLBI metabolic syndrome definition, an update of the NCEP ATP III, has the primary purpose for diagnosing the persons with metabolic syndrome for lifestyle therapies to reduce long-term risk of developing cardiovascular disease and type 2 diabetes mellitus. This issue has become more important since recent reports have suggested prevention/delay of diabetes with lifestyle intervention [45,46]. Thus, the stronger association between hs-CRP and metabolic syndrome by AHA/NHLBI definition in females indicates that lifestyle interventions should aim at female subpopulation with metabolic syndrome who will have a higher likelihood of development of diabetes and cardiovascular events.

In summary, the study demonstrates that elevated concentrations of hs-CRP showed a stronger association with metabolic syndrome in women than in men. This finding suggests that chronic inflammation may be of particular importance in the pathogenesis of metabolic syndrome in women. Our study finding has important implication on screening of metabolic syndrome.

## Competing interests

The authors declare that they have no competing interests.

## Authors' contributions

CSL, CCL and TCL contributed equally to the design of the study and direction of its implementation, including supervision of the field activities, quality assurance and control. CIL, CCC, WYL, MML, TL and PCC supervise the field activities. CSL and YDL helped conduct the literature review and prepare the Methods and the Discussion sections of the text. CSL, CCL, TCL and CIL designed the study's analytic strategy and conducted the data analysis. All authors read and approved the final manuscript.

## Pre-publication history

The pre-publication history for this paper can be accessed here:

http://www.biomedcentral.com/1471-2458/10/429/prepub
